# A Rare Case of Bartter Syndrome Type 3 Diagnosed in Elderly Age

**DOI:** 10.1155/crie/9463565

**Published:** 2026-06-09

**Authors:** Tsuyoshi Okura, Kenji Fukaya, Tetsuya Okazaki, Kaori Adachi, Yoshiharu Kinugasa, Masaru Kato

**Affiliations:** ^1^ Division of Cardiovascular Medicine, Endocrinology and Metabolism, Tottori University Faculty of Medicine, Yonago, Japan, tottori-u.ac.jp; ^2^ Division of Clinical Genetics, Tottori University Hospital, Yonago, Japan, tottori-u.ac.jp; ^3^ Department of Clinical Genomic Medicine, Graduate School of Medicine, Dentistry and Pharmaceutical Sciences, Okayama University, Okayama, Japan, okayama-u.ac.jp; ^4^ Research Initiative Center, Organization for Research Initiative and Promotion, Tottori University, Yonago, Japan, tottori-u.ac.jp

**Keywords:** Bartter syndrome, elderly, type 3

## Abstract

Bartter syndrome (BS) type 3 typically presents in childhood and is caused by defects in NaCl transporters of the thick ascending limb of the loop of Henle. We report a 66‐year‐old woman with asymptomatic but severe hypokalemia (2.0 mmol/L), metabolic alkalosis, and hyperreninemic hyperaldosteronism. Initial clinic blood pressure was 157/88 mmHg, but repeated office and home measurements were ~120/70 mmHg, consistent with white‐coat hypertension. Imaging excluded renovascular disease. Sequential diuretic testing supported BS physiology: a thiazide loading test increased fractional excretion of chloride (FECl) from 0.46% to 3.88% (ΔFECl 3.4%), exceeding the 2.3% cutoff that argues against Gitelman syndrome; a furosemide test showed chloride reabsorption of 10.1%, indicating a profound loop‐segment defect, even lower than values commonly reported in BS3. Targeted next‐generation sequencing identified a homozygous CLCNKB stop‐gain variant (c.1830G >A; p.Trp610Ter), confirming BS type 3. High‐dose potassium chloride (9.9 g/day) failed to correct hypokalemia, whereas low‐dose enalapril plus spironolactone normalized serum potassium (4.0 mmol/L) without persistent hypotension. The urine calcium/creatinine ratio was borderline‐normal (0.65 mmol/mmol), rather than hypocalciuric. This case suggests that BS3 should remain in the differential diagnosis of refractory hypokalemia in older adults and highlights the diagnostic value of physiology‐guided diuretic testing combined with genetic analysis, as well as the potential efficacy of low‐dose ACE inhibitor plus mineralocorticoid receptor antagonist therapy when potassium supplementation alone is insufficient.

## 1. Introduction

Bartter syndrome (BS) comprises autosomal‐recessive salt‐losing tubulopathies caused by defects in NaCl transporters of the thick ascending limb of the loop of Henle (e.g., NKCC2, ROMK, ClC‐Kb) and typically presents with hypokalemia and metabolic alkalosis [1]. In contrast, Gitelman syndrome (GS) results from loss of function of the sodium‐chloride cotransporter (NCC; SLC12A3) in the distal convoluted tubule [2]. BS subtypes (I, II, III, IV, and IVb) are defined by the causative gene, with type III due to CLCNKB variants [3].

Although BS most often manifests in infancy or childhood, type 3 (CLCNKB‐related) is relatively more likely to be identified first in adolescence or adulthood owing to its broader phenotypic range and milder early symptoms [3].

## 2. Case Presentation

A 66‐year‐old Japanese woman presented with severe hypokalemia detected on routine laboratory testing. She had no symptoms in childhood. She had been diagnosed with hypercholesterolemia at age 60 during routine health screening. She had been taking a statin since age 60. At age 65, severe hypokalemia (2.0 mEq/L [2.0 mmol/L]) was detected, prompting referral for further evaluation.

### 2.1. Diagnostic Assessment

Her height was 150 cm and body weight was 53 kg. Blood pressure at the first visit was 157/88 mmHg with a heart rate of 116/min; however, on repeated office and home measurements after anxiety subsided, values were consistently around 120/70 mmHg, indicating white‐coat hypertension rather than sustained hypertension.

Serum biochemical results (Table 1) indicated hypokalemia with hypochloremic metabolic alkalosis, along with elevated plasma renin activity and aldosterone levels.

**Table 1:** Laboratory test results on a clinical test.

Complete blood count		Biochemical tests		Endocrinological tests	
White blood cell	4200/μL	Na (135–145 mEq/L; 135–145 mmol/L)	141 mEq/L (141.0 mmol/L)	Plasma renin activity (0.3–2.9 ng/mL/h)	9.9 ng/mL/h
Neutrophil	61.50%	K (3.5–5.0 mEq/L; 3.5–5.0 mmol/L)	2.0 mEq/L (2.0 mmol/L)	Plasma aldosterone (4.0–82.1 pg/mL; 8–45 pmol/L)	47.9 pg/mL (133 pmol/L)
Lymphocyte	32.80%	Cl (98–106 mEq/L; 98–106 mmol/L)	96 mEq/L (96.0 mmol/L)	ACTH (10–60 pg/mL/2.2–13.2 pmol/L)	23.0 pg/mL (5.06 pmol/L)
Monocyte	4.30%	Ca (8.5–10.5 mg/dL; 2.1–2.6 mmol/L)	9.9 mg/dL (2.47 mmol/L)	Cortisol (5–25 μg/dL/138–690 nmol/L)	17.3 μg/dL (477.31 nmol/L)
Eosinophil	0.70%	P (2.5–4.5 mg/dL; 0.81–1.45 mmol/L)	3.1 mg/dL (1.0 mmol/L)	TSH (0.4–4.0 μU/mL/14–143 pmol/L)	1.29 μU/mL (46.1 pmol/L)
Basophil	0.70%	Mg (1.7–2.3 mg/dL; 0.7–0.95 mmol/L)	1.8 mg/dL (0.74 mmol/L)	Free T4 (0.8–1.8 ng/dL/10.3–23.2 pmol/L)	1.65 ng/dL (21.2 pmol/L)
Red blood cell	4.97 × 106/μL	Urea nitrogen (7–20 mg/dL; 2.5–7.1 mmol/L)	10.8 mg/dL (3.8 mmol/L)		
Hemoglobin	14.4 g/dL	Creatinine (0.6–1.2 mg/dL; 53–106 μmol/L)	0.59 mg/dL (52.2 μmol/L)	DHEAS (35–430 μg/dL/0.95–11.6 μmol/L)	107 μg/dL (2.95 μmol/L)
Platelet	29.0 × 104/μL	eGFR	77.0 mL/min/1.73 m^2^	Adrenaline (–0.17 ng/mL; –0.93 nmol/L)	0.09 ng/mL (0.491 nmol/L)
		Total protein (2.5–4.5 mg/dL)	7.0 g/dL	Noradrenaline (0.10–0.50 ng/mL; –0.59‐2.96 nmol/L)	0.63 ng/mL (3.72 nmol/L)
		Albumin	4.3 g/dL	Dopamine (−0.03 ng/mL; 0.196 nmol/L)	0.05 ng/mL (0.326 nmol/L)
Urinalysis		Total‐bilirubin (0.3–1.2 mg/dL; 5.1–20.5 μmol/L)	1.0 mg/dL (17.1 mmol/L)	U‐metanephrine (0.04–0.18 mg/day/0.20–0.92 μmol/L)	0.06 mg/day (0.306 μmol/day)
Protein	—	AST (10–40 U/L)	16 U/L	U‐normetanephrine (0.10–0.28 mg/day/0.55–1.54 μmol/L)	0.07 mg/day (0.384 μmol/day)
Glucose	—	ALT (7–56 U/L)	14 U/L		
Blood	—	ALP (44–147 U/L)	78 U/L	Arterial blood gas	
U‐Na	98 mmol/L	γ‐GTP (9–48 U/L)	18 U/L	pH	7.429
U‐K	11.8 mmol/L	LDH (140–280 U/L)	132 U/L	pCO_2_	41.6 mmHg
U‐Cl	86 mmol/L	CK (59–248 U/L)	63 U/L	pO_2_	85.2 mmHg
U‐Ca	3.5 mg/dL (0.87 mmol/L)			HCO_3_^−^	27.1 mmol/L
U‐Cr	15.0 mg/dL			BE	3.0 mmol/L

Abbreviations: ACTH, adrenocorticotropic hormone; DHEAS, dehydroepiandrosterone sulfate; TSH, thyroid stimulating hormone.

Renovascular hypertension was initially suspected. MR angiography demonstrated slight narrowing of the renal artery (Figure 1), whereas contrast‐enhanced CT revealed no significant renovascular stenosis (Figure 2). There was no significant decrease in blood flow at Doppler ultrasonography (Figure 3). There was also no significant decrease in blood flow on captopril renography (Figure 4). U waves were observed on electrocardiography, consistent with severe hypokalemia (Figure 5).

**Figure 1:**
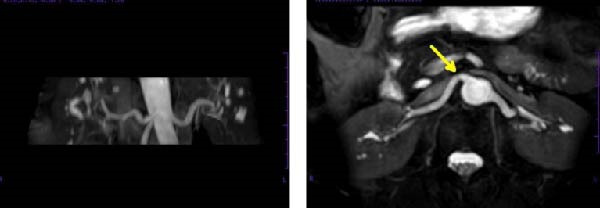
MR angiography. The arrow indicates a slight narrowing of the renal artery on MR angiography.

**Figure 2:**
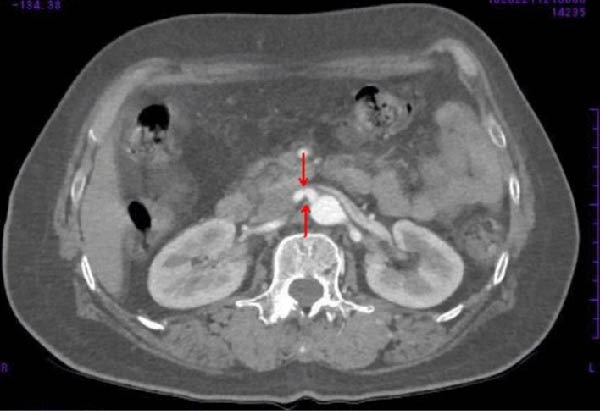
Enhanced CT. Arrows indicate that no significant renovascular stenosis was observed on contrast‐enhanced CT.

**Figure 3:**
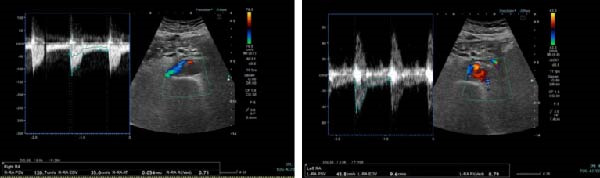
Doppler ultrasonography of the renal arteries. Doppler ultrasonography of the renal arteries showed no significant reduction in blood flow.

**Figure 4:**
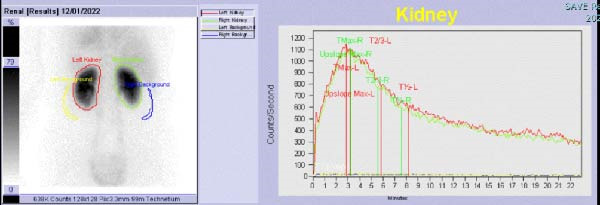
Captopril renogram. Captopril renography demonstrated no significant decrease in renal perfusion.

**Figure 5:**
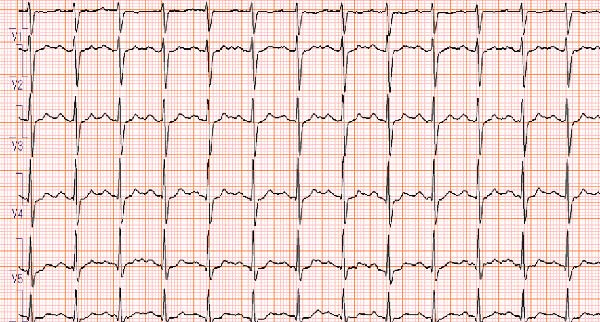
Electrocardiogram. Electrocardiography showed prominent U waves.

We planned further investigations to differentiate BS from GS according to established criteria [4].

The core biochemical features of BS include the following triad: (i) K^+^ <3.5 mmol/L, (ii) metabolic alkalosis, (iii) hyperreninemic hyperaldosteronism without hypertension. Supportive findings: urinary Cl^−^ >20 mmol/L, elevated urinary Ca^2+^ (except in type 3).

Physiology‐based tests: Where molecular testing is unavailable, sequential diuretic tests may help to separate BS from GS:

• Thiazide test: ΔFECl >2.3% favors BS.
• Furosemide test: chloride re‐absorption <20% strongly supports BS.

Consensus states these tests are not required when gene panels are accessible because of dehydration risk and limited specificity.

Molecular confirmation (gold standard): Perform a next‐generation sequencing panel for the five established BS genes: SLC12A1 (type I), KCNJ1 (II), CLCNKB (III), BSND ± CLCNKA/B (IVa/IVb), MAGED2 (transient neonatal type V). Detection of biallelic pathogenic variants confirms the diagnosis.

We performed a captopril challenge test; plasma renin activity increased appropriately and the test was negative (Table 2). The thiazide loading test increased FECl from 0.46% to 3.88% (ΔFECl 3.4%), which exceeds the 2.3% cutoff used to indicate preserved sodium–chloride cotransporter (NCC) function and therefore supports Bartter physiology rather than GS (Table 3). In contrast, the furosemide loading test yielded an estimated chloride reabsorption of 10.1%, indicating a profound loop‐segment defect—lower than commonly reported in BS3 cohorts (typically ~20%–65%) (Table 4). These sequential tests physiologically distinguish distal tubular NaCl transport dysfunction typical of GS from loop of Henle transporter defects characteristic of BS.

**Table 2:** Captopril challenge test.

Minutes	0	30	60	90	120
Plasma renin activity (ng/mL/h) (0.3–2.9 ng/mL/h)	53.7	35.0	32.3	50.4	37.5
Plasma aldosterone concentration (pg/mL) (4.0–82.1 pg/mL; 8–45 pmol/L)	16.5	50.8	37.2	68.4	65.7

**Table 3:** Thiazide challenge test.

Minutes	0	Pre‐load 30	Post‐load 30	60	90	120	150	180
FENa (%)	0.25	0.84	0.86	1.82	2.38	2.36	2.50	2.63
FECL (%)	0.46	1.46	1.44	2.81	3.32	3.29	3.56	3.88
Urinary volume (mL)	0	50	55	50	120	150	150	110
urine osmolarity (mOsm/kg·H_2_O)	416	313	152	146	163	177	217	253

*Note:* Delta FECL 3.8–0.4 = 3.4%, Delta FECL <2.3％ Gitelman.

Abbreviations: FECL, fractional excretion of chloride; FENa, fractional excretion of sodium.

**Table 4:** Furosemide challenge test.

Minutes	0	Pre‐load 20	Pre‐load 40	Post‐load 20	Post‐load 40	60	80	100	120
FENa (%)	1.07	1.08	1.90	7.64	14.1	10.3	7.16	6.06	4.39
FECL (%)	2.84	2.89	4.22	12.1	21.6	16.4	11.8	10.5	8.0
Urinary volume (mL)	50	50	50	200	230	150	190	70	110
urine osmolarity (mOsm/kg·H_2_O)	328	307	293	245	233	227	217	211	210

*Note:* Cl reabsorption 10.1%, normal 80%–98%, Gitelman 64%–88%, Bartter 20%–65%.

Abbreviations: FECL, fractional excretion of chloride; FENa, fractional excretion of sodium.

Spot urine calcium was 0.87 mmol/L (3.5 mg/dL) with urine creatinine 1.33 mmol/L (15 mg/dL), yielding a calcium‐to‐creatinine ratio of 0.65 mmol/mmol (0.23 mg/mg), which did not meet commonly used criteria for hypocalciuria (defined as <0.5 mmol/mmol).

According to these results, we considered BS versus GS, and we performed gene analysis. As a result of gene analysis, we found the variant of Bartter III syndrome (CLCNKB, NM_000085.5, homozygous, Stop_gained, c.1830G >A, p.Trp610Ter, Chr1:16055508, G/A) (Table 5). Although ClinVar associates c.1830G >A with both BS type 3 and type 4b, the lack of sensorineural deafness and BSND mutations in this patient supports classification as BS type 3.

**Table 5:** Gene analysis.

Variant No	Gene name	Feature ID	Genotype	Annotation	HGVS.c	HGVS.p	Position	Ref/Alt
1	*CLCNKB*	NM_000085.5	Homozygous	Stop_gained	c.1830G >A	p.Trp610Ter	Chr1:16055508	G/A
2	*CLCNKB*	NM_000085.5	Homozygous	Synonymous_variant	c.19717>C	p.Phe657 =	Chr1:16056463	T/C
3	*SLC12A1*	NM_000338.3	Heterozygous	Synonymous_varian	c.828G >A	p.Val276	Chr15:48229292	G/A
4	*CASR*	NM_000388.4	Heterozygous	Synonymous_varian	c.78C >G	p.Ala26 =	Chr3:122254267	C/G

### 2.2. Treatment (Figure 6)

High‐dose oral potassium chloride (9.9 g/day) failed to normalize serum potassium (Figure 6). Low‐dose enalapril was then initiated, increasing serum potassium to 3.2 mmol/L; however, systolic blood pressure decreased to 100 mmHg. Spironolactone alone was insufficient. Ultimately, combination therapy with enalapril (2.5 mg/day) and spironolactone (12.5 mg/day) stabilized serum potassium at 4.0 mmol/L without persistent hypotension.

**Figure 6:**
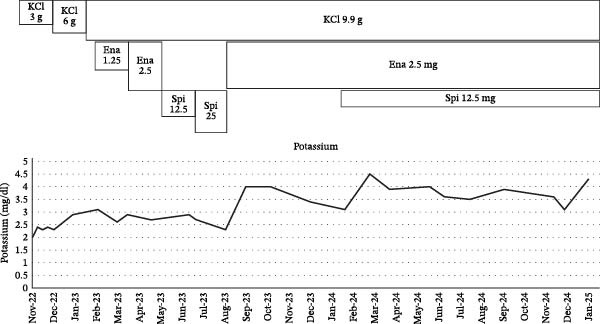
Time course of potassium. Ena, enalapril; KCl, potassium chloride; Spi, spironolactone.

### 2.3. Outcome and Follow‐up

Serum potassium remained stable with combination therapy consisting of enalapril (2.5 mg/day) and spironolactone (12.5 mg/day). This low‐dose combination approach may represent an effective therapeutic option when potassium supplementation alone is insufficient.

## 3. Discussion

Summary of the case—A 66‐year‐old woman with severe hypokalemia, metabolic alkalosis, and hyperreninemic hyperaldosteronism was ultimately diagnosed with BS type 3 (CLCNKB c.1830G >A, p.Trp610Ter). Sequential diuretic tests supported a loop‐segment defect (ΔFECl 3.4% on thiazide; chloride reabsorption 10% on furosemide), and low‐dose ACE inhibitor plus spironolactone normalized potassium when high‐dose KCl failed. The initially elevated blood pressure was attributable to white‐coat hypertension, resolving the apparent paradox with the biochemical profile.

### 3.1. Related Cases (Concise Summaries)

Related previously published cases are summarized narratively below; detailed structured data are provided in Table S1.

Tamagawa et al. [5] reported a 72‐year‐old man with long‐standing isolated ACTH deficiency who was ultimately diagnosed with BS type 3. Because of adrenal insufficiency, both diuretic loading tests were performed using a reduced‐dose protocol. The patient showed a good response to furosemide, whereas the response to thiazide was only mild. Urinary calcium excretion was within the normal range. Genetic testing demonstrated a homozygous CLCNKB c.1830G >A (p.Trp610Ter; W610X) variant. Potassium supplementation combined with glucocorticoid replacement improved biochemical control. The authors emphasized that this case exhibited several atypical features for BS type 3, including a late and mild clinical presentation suggestive of GS and nonclassical diuretic responses, highlighting diagnostic ambiguity at presentation. This elderly presentation with normal—not low—urinary calcium and mixed diuretic responses illustrates the phenotypic breadth of BS3 (see Table S1).

Chiang et al. [6] described a 45‐year‐old woman with classic Bartter physiology who experienced recurrent hypokalemic paralysis. Formal thiazide or furosemide loading tests were not reported. According to the available abstract, biochemical evaluation demonstrated hypercalciuria, with a urine calcium‐to‐creatinine ratio of 0.5 mmol/mmol. Compound heterozygous CLCNKB variants (L335P/G470E) were identified, and serum potassium levels improved after the addition of spironolactone to potassium supplementation. This case illustrates that adult‐onset BS3 may present with severe hypokalemia and hypercalciuria (see Table S1).

Mou and Wu [7] described an inbred Chinese pedigree in which the proband, a 48‐year‐old man, showed an overlapping BS–GS phenotype with hypomagnesemia and hypocalciuria. Diuretic loading tests were not described. Urinary calcium handling varied by genotype within the family: individuals with dual homozygous variants in SLC12A3 (c.248G >A, p.Arg83Gln) and CLCNKB (c.1171T >C, p.Trp391Arg) showed GS‐like hypocalciuria, whereas a relative with homozygous CLCNKB alone exhibited BS‐like hypercalciuria. Treatment included potassium/magnesium supplementation and mineralocorticoid receptor antagonism, underscoring marked intrafamilial heterogeneity across the BS–GS spectrum.

Stevenson et al. [8] reported a patient who had been clinically diagnosed with GS in childhood (at 10 years of age) based on phenotype alone, but was reclassified as BS type 3 in adulthood (at 55 years of age) following whole‐genome sequencing, which identified pathogenic variants in CLCNKB. Diuretic loading tests were not described; the report focused on genomic re‐evaluation alongside evolving clinical data. She subsequently developed hypocalciuria, further supporting an overlapping BS–GS phenotype. Treatment consisted of potassium and magnesium supplementation, with the addition of amiloride. This case illustrates how adult genomic reassessment can correct legacy labels (see Table S1).

These case reports collectively highlight the diagnostic difficulty of distinguishing BS3 from GS based on phenotype alone, particularly in adults. Precise diagnosis of inherited salt‐losing tubulopathies (SLTs) based on clinical and biochemical findings remains challenging even for experienced clinicians [9]. For example, the genetic confirmation rate among adult patients clinically diagnosed with GS was only 46% [10]. In this context, performing both thiazide and furosemide loading tests was clinically justified in the present patient.

Nozu et al. [11] performed pharmacologic typing in a clinical series of 16 genetically defined SLTs patients (BS types I–III and GS), all of whom underwent standardized furosemide and thiazide loading tests. As expected, NKCC2 defects showed no response to furosemide, and NCC defects showed no response to thiazide; however, substantial overlap was observed—particularly between CLCNKB (BS3) and SLC12A3 (GS)—such that these disorders can be difficult to distinguish in some patients even with diuretic challenge. This study supports combining diuretic testing with molecular analysis when BS3 is considered.

Fukuyama et al. [12]. reported two Japanese boys clinically diagnosed as classic BS who nonetheless exhibited hypocalciuria. No formal thiazide or furosemide loading tests were described; the report focused on biochemistry and genetics. Urine calcium excretion was low (urine Ca/creatinine molar ratio 0.07 and 0.03), and sequencing identified CLCNKB variants including W610X (with L27R in patient 1 and an intron‐2 splice‐site change in patient 2). These data provided early evidence that BS3 can present with hypocalciuria, although our patient did not demonstrate this feature.

### 3.2. Urinary Calcium Phenotype

In our patient, spot urinary calcium was 0.87 mmol/L (3.5 mg/dL) with a urine Ca/Cr ratio of 0.65 mmol/mmol, which is not consistent with hypocalciuria.

Classical BS subtypes associated with thick ascending limb of Henle’s loop (TAL) transporter defects (types I, II, IV, and V) frequently show hypercalciuria and nephrocalcinosis because impaired NKCC2/ROMK‐dependent salt transport reduces the lumen‐positive potential that normally drives paracellular Ca^2+^ reabsorption. By contrast, BS type 3 (CLCNKB) often exhibits normal‐to‐low urinary calcium, reflecting the unique expression of ClC‐Kb in both the TAL and the distal convoluted tubule: reduced distal NaCl reabsorption can mimic a Gitelman‐like physiology with enhanced proximal Ca^2+^ reabsorption and increased distal Ca^2+^ transport, thereby lowering urinary calcium.

Consistently, hypocalciuria has been documented in BS3 patients carrying CLCNKB stop‐gain variants, including Japanese cases [12], and adult cohorts emphasize that the urine Ca/Cr ratio is helpful for BS–GS differentiation even when phenotypes overlap [10]. Our patient’s values are consistent with the reported variability of urinary calcium handling in BS3.

In a clinical study of diagnosis of BS, GS, and pseudo–Bartter/GS in Japan, the mean age at diagnosis of Type III BS (*n* = 30) was 4.2 ± 14.0 years [3]. Patients with type III BS had a significantly lower mean age at diagnosis than did patients with GS or pseudo‐BS/GS (4.2 ± 14.0 vs. 18.0 ± 17.1 vs. 36.7 ± 16.5 years, respectively; *p* < 0.001). These findings underscore the rarity of late‐adulthood diagnosis of BS type 3. Therefore, BS3 should be considered in older patients presenting with refractory hypokalemia, even when childhood symptoms are absent.

This report has several limitations. First, it describes a single patient. Because both parents were deceased, segregation analysis was not possible. Although the patient has a younger sister and one son, neither exhibited hypokalemia or related symptoms; therefore, genetic testing was not pursued in these asymptomatic family members. However, phenotypic variability—including the presence or absence of hypokalemia—has been reported even among individuals carrying the same pathogenic variant within a single pedigree of inherited SLTs. Thus, the absence of symptoms is insufficient to exclude SLTs in at‐risk relatives. In addition, copy‐number variation analysis was not performed. Nevertheless, the identification of a homozygous stop‐gain CLCNKB variant, together with compatible physiology and the absence of features suggestive of BS type IV, strongly supports the diagnosis of BS type 3.

Despite these limitations, we believe that this case contributes meaningfully to clinical practice in endocrinology by highlighting the importance of considering BS type 3 in elderly patients with refractory hypokalemia.

In conclusion, this case highlights that BS type 3 can remain unrecognized until late adulthood and should be considered in older patients with refractory hypokalemia, particularly when physiological testing and genetic analysis indicate loop‐segment dysfunction.

### 3.3. Learning Points

BS type 3 should be considered in elderly patients with unexplained or refractory hypokalemia.

Sequential diuretic testing (thiazide and furosemide) combined with genetic analysis helps distinguish Bartter from GS.

A homozygous stop‐gain mutation in CLCNKB (c.1830G >A (p.Trp610Ter)) confirmed the diagnosis in this patient.

Low‐dose combination therapy with an ACE inhibitor and a mineralocorticoid receptor antagonist effectively normalized serum potassium when high‐dose potassium supplementation failed.

## Author Contributions

All authors made individual contributions to authorship. Tsuyoshi Okura, Kenji Fukaya, Tetsuya Okazaki, Kaori Adachi, Yoshiharu Kinugasa, and Masaru Kato were involved in the diagnosis and management of the patient and manuscript submission.

## Funding

There is no public or commercial funding.

## Disclosure

All authors reviewed and approved the final draft.

## Consent

Signed informed consent was obtained directly from the patient.

## Conflicts of Interest

The authors declare no conflicts of interest.

## Supporting Information

Additional supporting information can be found online in the Supporting Information section.

## Supporting information


**Supporting Information** Table S1: Provides a summary of previously reported adult‐onset Bartter syndrome type 3 cases, including clinical characteristics, genetic findings, and treatment outcomes.

## Data Availability

The data that support the findings of this study are available from the corresponding author upon reasonable request.
