# Supplementary material for: A Rare Case of Bartter Syndrome Type 3 Diagnosed in Elderly Age
Source: Case Rep Endocrinol. 2026 Jun 9;2026:9463565. doi: 10.1155/crie/9463565 (PMC13248106; doi:10.1155/crie/9463565)
Supplement: Supplementary file 1 — Supporting Information Table S1: Provides a summary of previously reported adult‐onset Bartter syndrome type 3 cases, including clinical characteristics, genetic findings, and treatment outcomes. [file CRIE-2026-9463565-s001.docx]

Supplementary Table S1. Summary of related adult BS/GS-spectrum cases (Present case + Refs. 5–8)

| Ref | Author (Year) | Age/Sex | Genotype | Thiazide test | Furosemide test | Urine Ca/Cr | Treatment | Outcome/Notes |
| --- | --- | --- | --- | --- | --- | --- | --- | --- |
| Present | Okura (2025, this report) | 66/F | CLCNKB c.1830G>A (p.Trp610Ter) homozygous | ΔFECl 3.4% (0.46→3.88%) | Chloride reabsorption 10.1% | 0.65 mmol/mmol (0.23 mg/mg) | KCl ineffective; + low‑dose ACE inhibitor + spironolactone | Normokalaemia (4.0 mmol/L) without hypotension |
| [5] | Tamagawa (2014) | 72/M | CLCNKB W610X homozygous | mild response | good response | U‑Ca/Cr 0.11 mg/mg; U‑Ca 0.064 g/day; FECa 1.63% | Potassium supplementation; glucocorticoid replacement | Elderly-onset BS type 3 complicated by isolated ACTH deficiency |
| [6] | Chiang (2014) | 45/F | CLCNKB L335P/G470E (compound het.) | Not reported (abstract only) | Not reported (abstract only) | Hypercalciuria  U‑Ca/Cr 0.5 mmol/mmol (normal<0.22 mmol/mmol) | KCl; + spironolactone | Fewer paralysis episodes |
| [7] | Mou & Wu (2021) | 48/M (index) | SLC12A3 + CLCNKB (dual homozygous) | Not reported | Not reported | Low in index (GS-like); varied by genotype within family | K/Mg supplementation; MRA | Intrafamilial heterogeneity with blended BS–GS phenotypes |
| [8] | Stevenson (2022) | 55/F  (10 years childhood GS) | CLCNKB on WGS | Not reported | Not reported | Mixed over time; GS‑like early | K/Mg; amiloride | Reclassified to BS3 after ~50 yrs |

Note: Data for Ref. [6] are derived from the abstract only due to limited full-text access at the time of revision.

MRAmineralocorticoid receptor antagonist:
